# Effect of a 12-Week Hybrid Mode Exercise Protocol on Obesity Indices and Health-Related Quality of Life in Diabetic Dyslipidemia

**DOI:** 10.7759/cureus.112094

**Published:** 2026-07-05

**Authors:** Janhavee S Brahmadande, Poovishnu T Devi, Rushikesh S Patil, Kiran S Dhaygude

**Affiliations:** 1 Department of Cardiopulmonary Sciences, Krishna College of Physiotherapy, Krishna Vishwa Vidyapeeth (Deemed to be University), Karad, IND

**Keywords:** central adiposity, diabetic dyslipidemia, health-related quality of life (hrqol), hybrid exercise protocol, metabolic health, obesity indices, type 2 diabetes mellitus

## Abstract

Background

Type 2 diabetes mellitus (T2DM) is a chronic metabolic condition defined by sustained elevated blood glucose and impaired insulin action, conditions that frequently give rise to diabetic dyslipidemia. This lipid disorder encompasses dangerous alterations in blood fat levels, most notably elevated triglycerides and depressed high-density lipoprotein (HDL) cholesterol, which together substantially heighten cardiovascular disease risk. Since abdominal adiposity amplifies these metabolic disturbances, structured lifestyle changes, especially exercise, are considered essential pillars of clinical management. The present investigation was designed to assess the effects of a 12-week hybrid exercise protocol combining aerobic and resistance modalities on obesity-related indices and health-related quality of life (HRQoL) in individuals with T2DM-associated dyslipidemia.

Objective

The study’s objective is to determine the effectiveness of a 12-week hybrid exercise protocol on obesity indices and HRQoL in diabetic dyslipidemia.

Study design and methodology

This randomized controlled trial was conducted over one year at Krishna College of Physiotherapy. Fifty-seven participants aged 45-60 years with T2DM-associated dyslipidemia and BMI >25 kg/m² were randomly allocated to a control group (n = 28) or an experimental group (n = 29). Outcomes included body weight, BMI, waist and hip circumference, waist-to-hip (WHR) ratio, suprailiac skinfold thickness, and Centers for Disease Control and Prevention (CDC) HRQoL-4 scores. Assessments were performed at baseline and after 12 weeks. The control group continued routine activities and walking, while the experimental group underwent a 12-week hybrid exercise protocol comprising aerobic, resistance, and flexibility exercises.

Results and comparative analysis

Pre-intervention assessments confirmed that both groups were broadly equivalent at baseline, though the experimental cohort displayed comparatively higher central adiposity and lower HRQoL scores. After completing the 12-week program, participants in the experimental group demonstrated statistically significant reductions across all obesity-related measures, including BMI (p < 0.0001), waist circumference (p < 0.0001), and WHR (p < 0.0001). Marked improvements were also recorded across all HRQoL domains, with notable declines in activity-limiting days and total unhealthy days (p < 0.0001 for most outcomes). Between-group comparisons validated that the hybrid exercise protocol yielded substantially greater benefits than standard management in both body composition and perceived quality of life.

Conclusion

These results establish that a 12-week hybrid exercise protocol is highly effective in improving metabolic parameters, decreasing central fat deposition, and promoting psychological well-being in patients with diabetic dyslipidemia. The observed benefits are likely attributable to the complementary mechanisms of aerobic exercise, which enhances fat oxidation, and resistance training, which preserves lean muscle and elevates metabolic efficiency. Future investigations should incorporate larger, multi-site cohorts and more comprehensive inflammatory biomarkers to evaluate whether these gains are sustained over the long term.

## Introduction

Type 2 diabetes mellitus (T2DM) is a long-standing metabolic disorder arising from progressive failure of pancreatic β-cell function and deteriorating insulin action, culminating in chronic hyperglycemia and widespread disruption of carbohydrate, protein, and fat metabolism. It represents the predominant form of diabetes globally and exerts a considerable burden on healthcare systems due to its rising prevalence and wide-ranging systemic complications [1]. Among the numerous metabolic sequelae associated with T2DM, diabetic dyslipidemia ranks among the most frequently encountered and is increasingly recognized as a primary driver of cardiovascular morbidity and mortality [2].

Diabetic dyslipidemia is pathophysiologically distinguished by hypertriglyceridemia, suppressed HDL cholesterol concentrations, and a preponderance of small, dense LDL cholesterol particles. These aberrations in lipid homeostasis stem primarily from insulin resistance, which disrupts the normal regulatory mechanisms of lipid metabolism and fosters an atherogenic milieu. Specifically, impaired insulin signaling accelerates lipolysis within adipose tissue, releasing an excess of free fatty acids into systemic circulation. The liver subsequently absorbs these fatty acids, driving heightened synthesis of triglycerides and very-low-density lipoprotein (VLDL) particles. Concurrently, diminished lipoprotein lipase activity compromises the catabolism of triglyceride-laden lipoproteins, compounding dyslipidemia [3]. Collectively, these disturbances markedly elevate the risk of cardiovascular disease and related metabolic complications in affected individuals [4].

The global burden of diabetic dyslipidemia has grown in parallel with rising rates of obesity and T2DM. Available evidence suggests that a majority of people with T2DM manifest at least one abnormal lipid parameter [5]. In India, this burden is particularly pronounced, with studies documenting lipid anomalies in upwards of 90% of diabetic patients [6]. Data drawn from the Indian Council of Medical Research-India Diabetes (ICMR-INDIAB) study further confirms the high prevalence of dyslipidemia among Indian adults, with depressed HDL-C identified as the most common abnormality [7]. Additionally, Parikh et al. documented a high occurrence and distinctive pattern of diabetic dyslipidemia in Indian individuals with T2DM, with combined dyslipidemia emerging as the most prevalent lipid phenotype [8].

Obesity constitutes one of the most consequential modifiable contributors to diabetic dyslipidemia. Visceral adiposity, particularly excess intra-abdominal fat, drives insulin resistance and interferes with lipid regulatory pathways [9]. Anthropometric indices such as BMI, waist circumference, and waist-to-hip (WHR) are routinely employed to quantify obesity-related health risk and have demonstrated robust associations with dyslipidemic patterns and cardiovascular risk burden [10].

Beyond its metabolic consequences, diabetic dyslipidemia imposes a substantial toll on health-related quality of life (HRQoL) [11]. Individuals managing both diabetes and dyslipidemia frequently report persistent fatigue, diminished physical capacity, emotional distress, and restrictions in daily activities. Research indicates that suboptimal glycemic and metabolic control correlates with lower HRQoL scores and a greater frequency of physically and mentally compromised days [12]. Dyslipidemia has similarly been linked with heightened disease burden and impaired psychosocial functioning [13]. The Centers for Disease Control and Prevention (CDC) HRQoL instrument (CDC HRQoL-4) is widely used in public health contexts to capture how individuals with chronic conditions perceive their physical and mental health, including the number of days affected by poor health [14].

Lifestyle modification remains a cornerstone of diabetic dyslipidemia management [15]. Exercise is recognized as among the most potent non-pharmacological strategies for enhancing metabolic health, improving lipid metabolism, and restoring insulin sensitivity [16]. Combined aerobic and resistance training regimens have demonstrated superior metabolic benefits compared with single-modality approaches [17]. Emerging evidence indicates that structured 12-week exercise programs can meaningfully improve cardiometabolic outcomes in individuals with dysglycemia and dyslipidemia [18].

Against this background, the present study was undertaken to evaluate the efficacy of a 12-week hybrid-mode exercise protocol on obesity indices and HRQoL in patients with diabetic dyslipidemia. The findings are anticipated to offer meaningful clinical insights into the role of comprehensive exercise-based interventions in improving physical health and overall quality of life in this high-risk population.

The present study aimed to evaluate the effect of a 12-week hybrid exercise protocol on obesity indices and HRQoL in individuals with diabetic dyslipidemia. The primary endpoint was change in obesity-related anthropometric measures, including body weight, BMI, waist circumference, hip circumference, WHR, and suprailiac skinfold thickness, while the secondary endpoint was change in HRQoL assessed using the CDC HRQoL-4 questionnaire. This study was undertaken to explore whether a structured hybrid exercise intervention could improve both obesity-related outcomes and overall quality of life in this high-risk metabolic population.

## Materials and methods

Study design

This study was designed as a randomized controlled trial, reported in accordance with Consolidated Standards of Reporting Trials (CONSORT) guidelines, to evaluate the effect of a 12-week hybrid exercise protocol on obesity-related indices and HRQoL in individuals with diabetic dyslipidemia. Participants were assigned to groups via simple random sampling. The study was conducted at Krishna College of Physiotherapy over a one-year period, including participant recruitment, baseline assessment, intervention, and post-intervention evaluation. The exercise intervention was administered for 12 weeks, from 6 November 2025 to 10 April 2026. Due to the nature of the exercise intervention, participant blinding was not feasible. Outcome assessment and statistical analysis were performed without formal blinding. A total of 57 eligible participants were enrolled and randomly allocated into a control group (Group A, n = 28) and an experimental group (Group B, n = 29). Owing to the nature of the exercise intervention, participant blinding was not feasible. No formal blinding of outcome assessors or statisticians was performed.

Randomization and allocation

After baseline assessment, participants were recruited using convenience sampling and randomly allocated by simple randomization. A total of 57 participants were allocated, with 28 participants in the control group and 29 participants in the experimental group. No formal allocation concealment procedure was employed.

Participants

A total of 57 participants were recruited through simple random sampling. Eligible participants included individuals of either sex between 45 and 60 years of age, carrying a diagnosis of T2DM-associated dyslipidemia, with a BMI exceeding 25 kg/m² and HbA1c ≥ 6.5%. Those with dyslipidemia attributable to other etiologies, recent bariatric surgery, ongoing pharmacological weight management, current participation in structured exercise programs, or unwillingness to provide consent were excluded. Written informed consent was obtained from all participants prior to study commencement, and the study received ethical clearance before initiation.

Outcome measures

Obesity Indices

Adiposity was quantified using standardized anthropometric methods encompassing body weight, BMI, and WHR. Body weight was recorded in kilograms using a calibrated weighing scale. BMI was derived by dividing body weight (kg) by height in meters squared (kg/m²) and served as an index for categorizing overweight and obese participants. Waist and hip circumferences were measured with a non-elastic tape, and WHR was computed by dividing waist by hip circumference. These measures are extensively employed to appraise both generalized and central adiposity and carry well-established associations with insulin resistance, T2DM, dyslipidemia, and elevated cardiovascular risk.

A BMI between 18.5 and 24.9 kg/m² is classified as normal weight, 25.0-29.9 kg/m² as overweight, and 30 kg/m² or more as obese. Elevated waist circumference and WHR are indicative of central obesity and heightened cardiometabolic risk. WHR is regarded as a sensitive marker of visceral adiposity and underlying metabolic dysfunction [19].

Suprailiac skinfold thickness was quantified using a calibrated skinfold calliper at the suprailiac site, positioned immediately superior to the iliac crest along the mid-axillary line. Measurements were recorded in millimeters and served as a surrogate for subcutaneous abdominal fat. Skinfold measurement is a straightforward, non-invasive, and reproducible method for appraising fat distribution and detecting changes in adiposity in response to exercise. Elevated suprailiac skinfold thickness reflects greater abdominal subcutaneous fat burden and increased susceptibility to obesity-related metabolic complications, while reductions in this measure signal improvements in body composition and fat loss [10].

Health-Related Quality of Life Scale

HRQoL was assessed using the CDC HRQoL-4 questionnaire developed by the CDC. It was selected as a brief and practical generic measure of overall health status, capturing general health, physically and mentally unhealthy days, and activity limitation, which aligned with the study objective of evaluating broad HRQoL changes following exercise intervention in individuals with diabetic dyslipidemia. This concise and widely adopted instrument is employed in population health surveillance to monitor well-being across communities. The core HRQoL-4 comprises four items evaluating self-rated general health, the number of days within the preceding 30 days during which physical health was poor, the number of days when mental health was impaired, and the number of days when ill health restricted habitual activities. These indicators assist researchers and clinicians in gauging disease burden, tracking health outcomes, and measuring the impact of therapeutic interventions on daily function and overall well-being.

Scores reflect the cumulative number of unhealthy days reported within the previous 30-day period. Physical and mental unhealthy days may be aggregated to yield a total unhealthy days score, representing the overall burden of compromised health. Higher scores denote more frequent days of poor physical or mental health and, correspondingly, lower HRQoL; lower scores reflect better perceived health and enhanced well-being. The instrument is well-suited for tracking quality of life changes in response to therapeutic interventions [14].

Intervention protocol

Control Group (Group A)

Participants in the control group were advised to continue their routine daily activities and walking throughout the 12-week study period. No structured supervised exercise protocol was administered to this group.

Experimental Group (Group B)

Participants in the experimental group underwent a 12-week hybrid exercise protocol comprising aerobic, resistance, and flexibility exercises. The intervention was administered five days per week, with each session lasting 50 minutes. Exercise intensity was progressively advanced from mild intensity in the initial phase to moderate intensity and subsequently high intensity according to participant tolerance over the intervention period. Sessions were conducted under supervision in the physiotherapy department.

Each exercise session consisted of a warm-up phase, aerobic exercise, resistance training, flexibility exercises, and a cool-down phase. The warm-up was performed for five minutes and included gentle mobility and stretching exercises. The aerobic component was performed for 20 minutes and included brisk walking. The resistance training component was performed for 15 minutes and included strengthening exercises for major upper- and lower-limb muscle groups using body-weight and resistance-based exercises. The flexibility component was performed for five minutes and included stretching exercises for the upper and lower extremities. A five-minute cool-down consisting of relaxation and stretching exercises was performed at the end of each session. Exercise intensity was progressively advanced from mild intensity in the initial phase to moderate and subsequently high intensity according to participant tolerance over the 12-week intervention period. All exercise sessions in the experimental group were supervised by the investigator/physiotherapist. Although participants were monitored during supervised sessions, a formal adherence analysis was not performed.

Procedure

The investigation was performed using a stepwise and methodical approach. Approval for conducting the research was first obtained from the institutional protocol committee to ensure adherence to ethical standards. Participants were subsequently enrolled after screening them according to the established eligibility criteria. Fifty-seven eligible participants were randomized into the control (n = 28) and experimental (n = 29) groups. All participants completed the 12-week intervention and post-intervention assessment. Therefore, complete outcome data were available for all randomized participants. The recorded observations were compiled and analyzed statistically, following which the findings were interpreted for the final outcome of the study.

Statistical analysis

Data were analyzed using SPSS software (IBM SPSS Statistics for Windows, IBM Corp., Armonk, NY). Normality of continuous variables was assessed using the Shapiro-Wilk test. Descriptive statistics were expressed as mean ± standard deviation for continuous variables and frequencies/percentages for categorical variables. Baseline characteristics between groups were compared using the independent t-test for continuous variables and the chi-square test for categorical variables. Within-group pre- to post-intervention changes were explored using paired t-tests for normally distributed variables and the Wilcoxon signed-rank test for non-normally distributed variables.

Because certain baseline differences were observed between the control and experimental groups, the primary between-group comparison of post-intervention outcomes was additionally performed using analysis of covariance (ANCOVA), with the post-intervention value as the dependent variable, study group as the fixed factor, and the corresponding baseline value as a covariate. Adjusted mean differences with 95% confidence intervals were reported. Effect sizes were calculated to estimate the magnitude of between-group differences. Statistical significance was set at p < 0.05.

All randomized participants completed the study and were included in the final analysis. Therefore, although the analysis followed the intention-to-treat principle, no imputation of missing data was necessary because there were no participant withdrawals or missing outcome data.

## Results

The study included 57 participants demonstrating a mean age of 52.40 ± 4.68 years, with a nearly equal distribution of 29 males (51%) and 28 females (49%). The participants were predominantly obese, as indicated by a mean BMI of 30.38 ± 1.89 kg/m² and a mean suprailiac skinfold thickness of 32.51 ± 3.24 mm. Anthropometric measurements showed a mean waist circumference of 98.05 ± 5.56 cm, hip circumference of 109.31 ± 5.87 cm, and WHR ratio of 0.897 ± 0.007, suggesting the presence of central obesity. HRQoL assessment revealed a mean general health score of 3.54 ± 0.87, with participants reporting 12.21 ± 5.67 physically unhealthy days and 8.35 ± 3.79 mentally unhealthy days. The mean unhealthy days score was 19.61 ± 7.36, while the healthy days score was 10.39 ± 7.36, indicating reduced quality of life. The calculated mean activity limitation score was 6.33 ± 4.83 days, reflecting the significance of diabetic dyslipidemia on daily functioning and overall well-being, as given in Table 1.

**Table 1:** Descriptive statistics of overall participants (n = 57) BMI - body mass index, HRQoL - health-related quality of life

Variables	Values
Age (mean ± SD)	52.4035 ± 4.68226
Sex (n) %	
Female	28 (49%)
Male	29 (51%)
BMI (mean ± SD)	30.38246 ± 1.885956
Suprailiac skinfold thickness	32.50878 ± 3.23553
HRQoL	
General health	3.54386 ± 0.86747
Physical health	12.2105 ± 5.66864
Mental health	8.35088 ± 3.7914
Unhealthy days	19.614 ± 7.35759
Healthy days	10.386 ± 7.35759
Activity limitation	6.333 ± 4.83046
Waist circumference	98.0474 ± 5.5587
Hip circumference	109.311 ± 5.87463
Waist-to-hip ratio	0.89684 ± 0.0066

The comparison between the non-intervention and experimental groups exhibited non-significant differences in age, sex distribution, BMI, suprailiac skinfold thickness, general health, mental health, activity limitation, and hip circumference (p > 0.05), signifying that both groups were equivalent and largely comparable at baseline. However, statistically relevant differences were observed in physical health (p = 0.0201), unhealthy days (p = 0.0002), healthy days (p = 0.0002), waist circumference (p = 0.0384), and WHR ratio (p = 0.0496). The experimental group reported poorer HRQoL and higher central obesity measures in contrast to the control group. Overall, while most demographic and obesity-related variables were comparable, certain HRQoL and central obesity parameters differed significantly between the groups at baseline, as given in Table 2.

**Table 2:** Comparison of baseline (pre-test) descriptive statistics between control and experimental group using chi-square test (χ²) for categorical data and independent t-test (t-value) for continuous variables BMI - body mass index, HRQoL - health-related quality of life

Variables	Control (n = 28)	Experimental (n = 29)	t/χ²	p-value
Age (mean ± SD)	51.82 ± 4.78	52.96 ± 4.59	-	-
Sex (n) %				
Female	14 (50%)	15 (52%)	-	-
Male	14 (50%)	14 (48%)	0.01694	0.8966
BMI (mean ± SD)	29.35 ± 1.188	29.882 ± 2.502	1.021	0.3118
Suprailiac skinfold thickness	34.607 ± 2.377	34.172 ± 2.904	0.6171	0.5397
HRQoL				
General health	3.535 ± 0.999	3.517 ± 0.508	0.08841	0.9299
Physical health	11.571 ± 6.999	14.758 ± 1.527	2.394	0.0201
Mental health	8.428 ± 4.795	10.103 ± 1.175	1.826	0.0734
Unhealthy days	18.357 ± 8.66	24.862 ±1.958	3.942	0.0002
Healthy days	11.642 ± 8.66	5.137 ± 1.958	3.942	0.0002
Activity limitation	5.785 ± 5.272	7.448 ± 2.472	1.533	0.1311
Waist circumference	96.021 ± 4.272	98.849 ± 5.66	2.121	0.0384
Hip circumference	106.521 ± 4.272	109.165 ± 6.980	1.717	0.0916
Waist-to-hip ratio	0.901 ± 0.0039	0.898 ± 0.005	2.007	0.0496

The within-group comparison of the control group showed no significant changes in BMI (p = 0.848), suprailiac skinfold thickness (p = 0.1100), waist circumference (p = 0.0544), and WHR ratio (p = 0.0570) following the intervention. Conversely, significant improvements were observed in several HRQoL domains, including general health (p = 0.0167), physical health (p = 0.0028), mental health (p = 0.0114), unhealthy days (p = 0.0097), healthy days (p = 0.0097), and activity limitation (p = 0.0114). A statistically significant shift was also seen in hip circumference (p = 0.0224). Overall, the non-intervention group demonstrated significant improvements in HRQoL, while obesity indices remained largely unchanged over the study period, as given in Table 3.

**Table 3:** Within-group comparison of pre-test and post-test data of the control group BMI - body mass index, HRQoL - health-related quality of life

Variables	Pre-test	Post-test	t-value	p-value
BMI (mean ± SD)	29.35 ± 1.188	29.289 ± 1.189	0.192	0.848
Suprailiac skinfold thickness	34.607 ± 2.377	34.392 ± 2.2	1.652	0.1100
HRQoL				
General health	3.535 ± 0.999	3.785 ± 0.686	2.553	0.0167
Physical health	11.571 ± 6.999	11.285 ± 6.798	3.286	0.0028
Mental health	8.428 ± 4.795	8.214 ± 4.685	2.714	0.0114
Unhealthy days	18.357 ±8.66	18 ± 8.476	2.285	0.0097
Healthy days	11.642 ± 8.66	12 ± 8.476	2.785	0.0097
Activity limitation	5.785 ± 5.272	8.214 ± 4.685	2.714	0.0114
Waist circumference	96.021 ± 4.272	95.039 ± 4.166	2.011	0.0544
Hip circumference	106.521 ± 4.272	106.503 ± 4.26	2.423	0.0224
Waist-to-hip ratio	0.901 ± 0.0039	0.900 ± 0.0035	1.988	0.0570

The within-group comparison of the intervention cohort exhibited statistically significant improvements in all outcome measures following the 12-week hybrid exercise protocol. Significant decreases were noted in BMI (p < 0.0001), suprailiac skinfold thickness (p < 0.0001), waist circumference (p < 0.0001), hip circumference (p < 0.0001), and WHR ratio (p < 0.0001), indicating substantial improvements in obesity indices and body fat distribution. Substantial improvements were also observed in all HRQoL domains, including general health (p < 0.0001), physical health (p < 0.0001), mental health (p = 0.0018), unhealthy days (p < 0.0001), healthy days (p < 0.0001), and activity limitation (p = 0.0011). Overall, the evidence suggests that the 12-week hybrid exercise protocol was highly effective in improving both obesity indices and HRQoL among individuals with diabetic dyslipidemia, as given in Table 4.

**Table 4:** Within-group comparison of pre-test and post-test data of the experimental group BMI - body mass index, HRQoL - health-related quality of life

Variables	Pre-test	Post-test	t-value	p-value
BMI (mean ± SD)	29.882 ± 2.502	28.0345 ± 1.822	5.096	<0.0001
Suprailiac skinfold thickness	34.172 ± 2.904	32.517± 3.066	18.427	<0.0001
HRQoL				
General health	3.517 ± 0.508	2.620 ± 0.493	6.666	<0.0001
Physical health	14.758 ± 1.527	8.517 ± 1.298	16.282	<0.0001
Mental health	10.103 ± 1.175	9 ± 1.388	3.458	0.0018
Unhealthy days	24.862 ±1.958	17.517 ± 2.487	12.868	<0.0001
Healthy days	5.137 ± 1.958	12.275 ± 2.250	13.370	<0.0001
Activity limitation	7.448 ± 2.472	5.586 ± 1.918	3.632	0.0011
Waist circumference	98.849 ± 5.66	92.413 ±5.401	32.625	<0.0001
Hip circumference	109.165 ± 6.980	103.862 ± 5.565	6.048	<0.0001
Waist-to-hip ratio	0.898 ± 0.005	0.889 ± 0.006	7.747	<0.0001

The post-intervention comparison intergroup analysis of control and experimental groups identified statistically significant differences in all outcome measures. The experimental cohort revealed significantly diminished change in BMI (p = 0.0033) and suprailiac skinfold thickness (p = 0.0106) versus the non-intervention group, indicating greater improvement in obesity indices. Significant reductions were also observed in waist circumference (p = 0.0400), hip circumference (p = 0.0497), and WHR ratio (p < 0.0001) in the experimental group. Regarding HRQoL, the intervention arm demonstrated significantly superior outcomes in general health (p < 0.0001), physical health (p = 0.0357), mental health (p = 0.0168), unhealthy days (p = 0.0373), healthy days (p = 0.0373), and activity limitation (p = 0.0092) against the control group. In summary, the findings show that the 12-week hybrid exercise protocol was more beneficial than routine daily activities and walking in improving obesity indices and HRQoL in individuals with diabetic dyslipidemia, as given in Table 5.

**Table 5:** Comparison of post-intervention values between the control group (n = 28) and the experimental group (n = 29) BMI - body mass index, HRQoL - health-related quality of life

Variables	Post-intervention mean		Mean difference	t-value	p-value
	Control	Experimental			
BMI (mean ± SD)	29.289 ± 1.189	28.0345 ± 1.822	-0.1254	3.067	0.0033
Suprailiac skinfold thickness	34.392 ± 2.2	32.517± 3.066	-1.876	2.645	0.0106
HRQoL					
General health	3.785 ± 0.686	2.620 ± 0.493	-1.165	7.377	<0.0001
Physical health	11.285 ± 6.798	8.517 ± 1.298	-2.768	2.153	0.0357
Mental health	8.214 ± 4.685	5.931± 0.842	-2.283	2.539	0.0168
Unhealthy days	18 ± 8.476	14.448 ±1.502	-3.552	2.1846	0.0373
Healthy days	12 ± 8.476	15.551 ± 1.502	3.551	-2.184	0.0373
Activity limitation	8.214 ± 4.685	5.586 ± 1.918	-2.628	2.7538	0.0092
Waist circumference	95.039 ± 4.166	92.413 ± 5.401	-2.626	-2.06	0.040
Hip circumference	106.503 ± 4.26	103.862 ± 5.565	-2.642	2.007	0.0497
Waist-to-hip ratio	0.900 ± 0.0035	0.889 ± 0.006	-0.006469	5.626	<0.0001

## Discussion

Diabetic dyslipidemia is a common metabolic abnormality in T2DM, characterized by elevated triglycerides, increased atherogenic lipoproteins, reduced HDL cholesterol, and insulin resistance. When accompanied by obesity, particularly visceral adiposity, it further increases cardiovascular risk and adversely affects overall health. Accordingly, interventions capable of addressing obesity, metabolic dysfunction, and physical deconditioning simultaneously are considered indispensable for comprehensive disease management [2,3].

The present investigation evaluated the effects of a 12-week structured hybrid exercise program comprising aerobic, resistance, and flexibility exercises on obesity indices and HRQoL in individuals with diabetic dyslipidemia. The findings demonstrated that participants assigned to the structured exercise intervention achieved significant improvements across multiple health-related domains when contrasted with those receiving routine care. Suder et al. [20] and Alghadir et al. [21] similarly documented that combined exercise regimens yield considerable gains in metabolic health and functional capacity in individuals with chronic metabolic conditions, lending further credence to the clinical relevance of exercise as a supportive intervention.

Feingold [10] described how aerobic activities increase caloric expenditure and enhance cardiovascular efficiency, while resistance training maintains or augments lean body mass and elevates basal metabolic activity. Chandana et al. [6] further observed that the concurrent application of these modalities creates conditions favorable to adiposity reduction, improved insulin responsiveness, optimized lipid utilization, and enhanced physical function. The concurrent improvements noted across multiple outcome measures in the present study support the notion that exercise exerts coordinated, system-wide physiological effects rather than discrete changes in single parameters.

BMI is among the most widely accepted and commonly utilized anthropometric indices for classifying obesity and gauging cardiometabolic risk. Elevated BMI is strongly correlated with insulin resistance, atherogenic dyslipidemia, hypertension, and cardiovascular disease, making it a clinically important therapeutic target in individuals with diabetic dyslipidemia.

Participants assigned to the hybrid exercise program demonstrated a marked reduction in BMI relative to the control group. Specifically, the intervention group showed a statistically significant decline in BMI from 29.88 ± 2.50 kg/m² to 28.03 ± 1.82 kg/m² (p < 0.0001), whereas the control group exhibited only a negligible and non-significant reduction from 29.35 ± 1.18 kg/m² to 29.29 ± 1.18 kg/m² (p = 0.848). Post-intervention between-group comparison showed a statistically significant difference favoring the experimental arm (p = 0.0033), suggesting that the hybrid protocol was associated with improvements in reducing obesity.

Suder et al. [20] and Shrivastava et al. [22] established that structured exercise produces meaningful reductions in obesity-related anthropometric parameters and improves overall body composition. The reductions observed in the current study reflect the cumulative benefits of sustained physical activity and signify successful weight management over the intervention period.

Feingold [10] explained that aerobic exercise elevates daily energy expenditure and promotes the mobilization and oxidation of stored lipids through enhanced lipolysis. 

Feingold [10] further highlighted that exercise-mediated improvements in insulin sensitivity lead to more efficient glucose utilization and reduced fat deposition, supporting sustained reductions in body mass and favorable changes in metabolic health. These findings are consistent with a broader body of evidence confirming that combined aerobic and resistance exercise effectively reduces obesity-related measures and cardiometabolic risk in individuals with metabolic disorders.

Schofield et al. [2] and Paduraru et al. [13] reported that reductions in BMI are associated with improved insulin sensitivity, more favorable lipid regulation, lower inflammatory burden, and reduced cardiovascular risk. The BMI reductions observed in the present study may therefore have contributed directly to the improvements noted in glycemic regulation, lipid profiles, and functional capacity.

Waist circumference is recognized as an important indicator of central adiposity and provides clinically meaningful information regarding regional fat distribution. Unlike generalized adiposity measures, waist circumference directly quantifies abdominal fat accumulation, particularly visceral adipose tissue, which is tightly linked to insulin resistance, metabolic syndrome, and cardiovascular disease risk.

In the present investigation, the experimental group demonstrated a highly significant reduction in waist circumference from 98.84 ± 5.66 cm to 92.41 ± 5.40 cm following the intervention (p < 0.0001). In contrast, the control group showed a non-significant reduction from 96.02 ± 4.27 cm to 95.03 ± 4.16 cm (p = 0.0544). Post-intervention intergroup comparison confirmed a statistically significant difference favoring the experimental group (p = 0.040), reinforcing the effectiveness of the hybrid protocol in reducing central adiposity.

Suder et al. (2024) reported that combined aerobic and resistance exercise programs reliably reduce abdominal adiposity and produce associated improvements in metabolic health. The magnitude of waist circumference reduction in the present study indicates that the intervention successfully targeted central obesity, a major driver of metabolic dysfunction [20].

Suder et al. [20] further explained that aerobic training promotes triglyceride breakdown from stored fat depots, while resistance exercise maintains lean muscle mass and sustains resting energy expenditure. These coordinated physiological adaptations collectively reduce abdominal fat accumulation over time.

Chandana et al. [6] and Feingold [10] reported that reductions in visceral fat improve peripheral insulin sensitivity and attenuate the release of inflammatory mediators that impair glucose metabolism. Consequently, the waist circumference reductions observed in the current study may have supported the improvements recorded in glycemic and lipid profile parameters.

Nandasena et al. [23] and Al Quran et al. [24] identified abdominal obesity as a powerful independent predictor of cardiovascular disease and systemic metabolic complications. The substantial reduction in waist circumference achieved by the experimental group may therefore signify a clinically meaningful reduction in future cardiovascular risk.

Hip circumference is a relevant anthropometric parameter reflecting regional body fat distribution and overall compositional change. In the current study, the experimental group demonstrated a statistically significant reduction in hip circumference from 109.16 ± 6.98 cm to 103.86 ± 5.56 cm (p < 0.0001), whereas the control group exhibited only a minimal change from 106.52 ± 4.27 cm to 106.50 ± 4.26 cm. Post-intervention comparison revealed a statistically significant advantage in favor of the experimental group (p = 0.0497).

Costa et al. [25] reported that structured exercise programs effectively modify regional body composition and reduce adipose tissue burden. The pronounced reduction in hip circumference in the present study reflects positive morphological adaptations to regular physical training.

Hirano [3] and Feingold [10] explained that repeated aerobic and resistance exercise bouts elevate energy expenditure, enhance lipid oxidation, and improve insulin sensitivity, collectively driving reductions in body circumference and favorable redistribution of body fat.

Prior investigations have similarly documented reductions in body circumferences following combined exercise regimens in individuals with metabolic disorders. These findings highlight that structured exercise exerts compositional benefits extending beyond simple reductions in body weight.

Paduraru et al. [13] noted that improvements in anthropometric measures may enhance mobility, reduce mechanical load on weight-bearing joints, and support improved metabolic function. The reduction in hip circumference observed in the present study should therefore be considered an additional dimension of the health benefits conferred by regular structured exercise.

WHR is widely accepted as a reliable measure of fat distribution and central adiposity. Elevated WHR values are robustly associated with insulin resistance, cardiovascular disease risk, and metabolic syndrome.

In the present study, the experimental group demonstrated a highly significant reduction in WHR from 0.898 ± 0.005 to 0.889 ± 0.006 (p < 0.0001), whereas the control group showed only minor and non-significant changes. Post-intervention intergroup analysis confirmed a highly significant difference between groups (p < 0.0001).

Suder et al. [20] and Zhou et al. [26] highlighted that regular physical activity and combined exercise interventions effectively reduce central adiposity and improve metabolic profiles. The substantial WHR reduction observed in the present study reflects a favorable redistribution of body fat in response to the intervention.

Feingold [10] explained that visceral adipose tissue is readily mobilized by exercise-induced hormonal shifts that stimulate lipolysis. As abdominal fat depots diminish, waist circumference decreases more rapidly than hip circumference, resulting in lower WHR values and a more favorable fat distribution pattern.

Nandasena et al. [23] and Al Quran et al. [24] emphasized that reductions in WHR are associated with decreased cardiovascular morbidity and improved metabolic health outcomes. The WHR improvements observed in the current study may therefore represent a clinically meaningful reduction in future cardiometabolic risk.

Suprailiac skinfold thickness serves as a direct measure of subcutaneous fat deposition and is routinely employed to track changes in body composition. In the present study, the experimental group showed a highly significant reduction in suprailiac skinfold thickness from 34.17 ± 2.90 mm to 32.51 ± 3.06 mm (p < 0.0001), while only minor changes were noted in the control group. Post-intervention analysis confirmed a significant between-group difference (p = 0.0106).

Suder et al. [20] and Costa et al. [25] reported that combined aerobic and resistance exercise effectively reduces subcutaneous fat stores and improves body composition. The reduction in suprailiac skinfold thickness in the present study indicates successful mobilization of adipose tissue reserves in response to training.

Hirano [3] and Chandana et al. [6] similarly documented significant reductions in skinfold measurements following structured exercise programs, corroborating the effectiveness of combined exercise in reducing both subcutaneous and visceral adiposity.

Feingold [10] emphasized that decreases in body adiposity contribute directly to improvements in insulin sensitivity, lipid profiles, and chronic low-grade inflammation. The reductions in suprailiac skinfold thickness observed in the current study may therefore have played a contributory role in the favorable metabolic changes recorded in glycemic and lipid.

Alghadir et al. [21] further explained that exercise positively modulates mental well-being through increased endogenous production of endorphins and mood-regulating neurotransmitters. Engagement in structured exercise programs also fosters self-efficacy, motivation, and psychological confidence, collectively enhancing emotional health and general quality of life in individuals managing chronic metabolic disorders.

Although the experimental group demonstrated greater post-intervention improvements across multiple outcomes, these findings should be interpreted in light of the baseline imbalance observed between groups, which may have influenced between-group comparisons.

Strengths

The present study incorporated several features that strengthened its clinical relevance and scientific quality. The randomized controlled design improved internal validity and mitigated selection bias. The use of multiple outcome measures spanning anthropometric, glycemic, lipid, functional, and quality of life domains provided a comprehensive characterization of intervention effects. The hybrid exercise protocol, combining aerobic and resistance training and flexibility exercises, aligns with current evidence-based exercise recommendations for individuals with diabetic dyslipidemia. The 12-week duration was sufficient to elicit measurable physiological adaptations, while the inclusion of functional performance and HRQoL endpoints added clinically meaningful dimensions beyond standard laboratory measurements. Standardized assessment procedures enhanced data reliability, and the deliberate recruitment of a high cardiovascular risk population increased the clinical significance of the findings.

Limitations

The present study has several limitations. Baseline imbalance in selected variables was observed between groups, which may have affected between-group comparability. Owing to the nature of the exercise intervention, participant blinding was not feasible, and formal allocation concealment as well as blinding of outcome assessors and statisticians were not performed, which may have introduced performance and assessment bias. Dietary intake, medication use, and physical activity outside the intervention sessions were not standardized or systematically monitored during follow-up, and any changes in medication may have influenced the outcomes. Adherence to the prescribed exercise protocol was not formally analyzed. In addition, the relatively small sample size and single-center design limit the generalizability of the findings. The 12-week intervention period did not permit evaluation of the long-term sustainability of the observed effects, and advanced biomarkers of inflammation and insulin resistance, such as CRP, IL-6, and homeostasis model assessment of insulin resistance (HOMA-IR), were not included. A formal a priori sample size calculation was also not performed, which may affect the precision of the estimated treatment effects. These factors should be addressed in future multicenter studies with larger samples, longer follow-up, and more comprehensive metabolic and behavioral monitoring.

Future directions

Based on the current findings and acknowledged limitations, several recommendations for future research can be advanced. Subsequent studies should recruit larger, multi-center samples to enhance external validity. Longer intervention periods with extended follow-up assessments are recommended to determine the long-term sustainability of exercise-related benefits. Trials integrating nutritional interventions alongside exercise protocols may offer a more holistic approach to managing diabetic dyslipidemia. Inclusion of comprehensive biomarkers such as CRP, IL-6, HOMA-IR, and detailed body composition assessments would provide deeper insight into underlying physiological mechanisms. Comparative investigations exploring varying exercise intensities, frequencies, and modalities are also warranted. Finally, future work should examine long-term adherence strategies and the viability of tele-rehabilitation and home-based exercise delivery models to improve accessibility and continuity of care.

## Conclusions

The present study suggests that a 12-week hybrid exercise protocol was associated with improvements in obesity indices and HRQoL in individuals with diabetic dyslipidemia. Compared with the control group, the experimental group demonstrated greater improvements following the intervention period. However, these findings should be interpreted with caution due to baseline group imbalances, the relatively small sample size, short follow-up duration, lack of blinding, and absence of standardized monitoring of diet and medication use. Further multicenter studies with larger samples and longer follow-up are needed to confirm these findings.
